# Synergistic apoptosis of human gastric cancer cells by bortezomib and TRAIL

**DOI:** 10.7150/ijms.34398

**Published:** 2019-09-20

**Authors:** Hang Thi Thuy Bui, Nhu Huynh Le, Qui Anh Le, Sung Eun Kim, Sooho Lee, Dongchul Kang

**Affiliations:** 1 Ilsong Institute of Life Science, Hallym University, Anyang, Kyonggi-do, 14066, Republic of Korea.; 2 Department of Biomedical Gerontology, Hallym University Graduate School, Chuncheon, Kangwon-do, 24252, Republic of Korea.; 3 Department of Internal Medicine, Hallym University Sacred Heart Hospital, College of Medicine, Hallym University, Anyang, Kyonggi-do, 14068, Republic of Korea.

**Keywords:** Gastric cancer, TRAIL, Bortezomib, DR4, DR5, ERK, p21^cip1/waf1^

## Abstract

Resistance against tumor necrosis factor-related apoptosis-inducing ligand (TRAIL)-induced cell death of cancer cells is a major obstacle in clinical application of TRAIL. Variable response to TRAIL of gastric cancer cells, synergy of TRAIL with bortezomib and potential mechanisms behind the phenomena were investigated in this study. The response to TRAIL varied among six gastric cancer cell lines, which correlated with the expression of apoptotic TRAIL receptors. Analysis of TCGA gene expression data showed that DR4 expression correlated with DR5 in gastric cancer. Although higher expression of DR4 was significantly associated with lower T, N and TNM stages, neither DR4 nor DR5 expression meaningfully influenced overall survival rate. Combined treatment of TRAIL with bortezomib resulted in strong synergistic response with enhanced activation of caspases-8, -9 and -3, and increased Annexin V-binding cell fractions in TRAIL-resistant SNU-216 cells. Bortezomib increased the expression of p21^cip1/waf1^, but p21^cip1/waf1^ silencing did not restore cell viability significantly. Bortezomib also increased DR5 expression and knockdown of DR5 expression significantly recovered cell viability reduced by the combination treatment. Bortezomib decreased phosphorylation of ERK1/2, but increased that of JNK. Treatment with either an ERK1/2 inhibitor U0126 or a JNK inhibitor SP600125 rescued SNU-216 from dying of bortezomib or combined treatment. However, upregulation of DR5 by bortezomib was knocked down only by inhibition of ERK1/2 activation significantly, but not by JNK activity inhibition. In summary, upregulation of DR5 by bortezomib is of critical significance in the synergy of bortezomib with TRAIL in apoptosis of TRAIL-resistant SNU-216 and that activity of ERK1/2 is required in the bortezomib-induced DR5 overexpression.

## Introduction

Gastric cancer is the third leading cause of cancer death in the world and half of the total cases occur in East Asia, particularly in China, Japan and Korea 1. Almost one million new cases of gastric cancer were diagnosed each year (6.8% of the total), making it the fifth common malignancy in the world according to GLOBOCAN 2012 2. Mortality by gastric cancer has been decreased by advances in diagnosis, surgery and novel treatment regimens, but the prognosis of the patients with advanced gastric cancer still remains poor 3.

The defect in apoptosis is a causative factor of tumorigenesis, tumor metastasis and anticancer drug resistance 4. Tumor necrosis factor (TNF)-related apoptosis-inducing ligand (TRAIL) induces apoptotic cell death in various cancer cell types including breast, bladder, lung, liver and stomach cancers, whilst generally sparing non-malignant cells 5, 6. The selective cytotoxicity of TRAIL against cancer cells has gained an intense interest in exploring the potential utility of TRAIL as an anticancer therapeutics 5. However, resistance to TRAIL-induced apoptosis of various cancer cells has been reported in numerous cases 6, 7. TRAIL-induced apoptosis can be inhibited by diverse mechanisms such as blocking death inducing signalling complex (DISC) formation, overexpression of anti-apoptotic proteins such as Bcl-2 and Bcl-X_L_ and inhibition of active caspases by IAPs. The exact mechanism of the resistance to TRAIL-induced apoptosis varies depending on cell types and has not been fully understood, yet. Drug combination with conventional and targeted cancer therapeutics is most widely attempted to overcome the TRAIL resistance 7, 8.

In this study, we attempted to determine differential response to TRAIL-induced apoptosis of human gastric cancer cells and to identify potential indicators of the TRAIL response. An association of the expression of DR4 and DR5 with clinicopathological phenotypes of gastric cancer patients was also analyzed with TCGA (The Cancer Genome Atlas) data. TRAIL-induced apoptosis of gastric cancer cells was enhanced by combined treatment with various reagents including 5-fluorouracil, cisplatin, paclitaxel and bortezomib 9, 10. Therefore, we have examined whether combined treatment of TRAIL with conventional chemotherapeutics can overcome the TRAIL resistance of the gastric cancer cells and what would be a potential mechanism underlying the synergistic induction of apoptosis.

## Materials and methods

### Gastric cancer cell lines and cell culture

The human gastric cancer cell lines, SNU-216, SNU-484, SNU-601, SNU-638, SNU-668 and SNU-719 were purchased from Korea Cell Line Bank (Seoul, Korea). They were cultured in RPMI-1640 (Gibco, Grand Islands, NY, USA) supplemented with 10% fetal bovine serum (Welgene, Daegu, Korea), 5% L-Glutamine (Gibco), 5% penicillin/streptomycin (Gibco) and maintained at 37^°^C in a humidified 5% CO_2_ atmosphere.

### MTT assay

The viability of cells was measured by 3-(4,5-dimethylthiazol-2-yl)-2,5-diphenyltetrazolium bromide (MTT) assay. Cells were seeded in 96-well plates at density of 8 x 10^3^ cells per well in 100 µl culture medium (RPMI-1640) one day prior to the treatment. Cells were treated with reagents as specified in figure legends. MTT (5 mg/ml MTT in PBS, Sigma-Aldrich, St. Louis, MO, USA) was treated and left for 3 h, and solubilized as previously described 11. The absorbance at 570 nm with reference absorbance at 650 nm was measured using a Multiskan^TM^ GO spectrophotometer (Thermo Scientific, Rockland, IL, USA). Results were calculated by subtracting blank readings, in which cells were not seeded.

### Western blot analysis

Cells were lysed in RIPA buffer (50 mM Tris-HCl pH 7.4, 0.1% SDS, 1% Triton X-100, 0.1% Nonidet P-40, 0.5% sodium deoxycholate, 1 mM DTT and protease inhibitors). Protein amount was estimated by BCA Protein Assay Reagent (Pierce, Rockford, IL, USA). Equal amounts of protein samples (25 µg/lane) were resolved by 10% or 12% SDS-polyacrylamide gel electrophoresis (PAGE) and transferred to a nitrocellulose membrane (PALL Corporation, Port Washington, NY, USA). Immunodetection except visualization was performed as previously described 11. ECL-treated blots (Advansta, Menlo Park, CA, USA) were exposed to a ChemiDoc^TM^ MP System (BioRad, Hercules, CA, USA) to visualize specific proteins. Intensity of detected bands was analyzed with Image J software (ij152-win-java8 downloaded from https://imagej.nih.gov/ij/) and quantification results are provided in Supplemental Figures. Antibodies used in the western blotting were anti-DR4 (Abnova Corporation, Taipei, Taiwan), anti-DR5, anti-p21^cip1/waf1^, anti-caspase-8, anti-caspase-9, anti-caspase-3, cleaved anti-caspase-8, cleaved anti-caspase-9, cleaved anti-caspase-3, anti-FLIP, anti-XIAP, anti-Bid, anti-Puma, (Cell Signaling Tech., Danvers, MA, USA), anti-BAX, anti-p53, anti-ERK, anti-phospho-ERK, anti-JNK, anti-phospho-JNK, anti-cIAP2, anti-β-actin (Santa Cruz Biotech., Dallas, Texas, USA), anti-p38 MAPK (ABM, Richmond, BC, Canada), and anti-phospho-p38 MAPK (Chemicon, Temecula, CA, USA).

### Flow cytometric analysis

Cells were seeded in 6-well plates at density of 5 x 10^5^ cells per well one day prior to treatment with indicated drugs. At specific time point, cells were harvested by trypsinization. The collected cells were washed once with 1X Annexin V binding buffer (eBioscience, San Diego, CA, USA) and then incubated in the buffer containing FITC-conjugated Annexin V (eBioscience). After incubated for 30 min at room temperature in dark, the cells were washed once with binding buffer and resuspended in 500 µl binding buffer containing propidium iodide solution (PI, 0.5 μg/ml). Annexin V binding and PI infiltration were evaluated by flow cytometry using a FACSCalibur™ (BD Bioscience, Sparks, MD, USA) and analyzed with CellQuest Pro™ software (BD Bioscience). To measure receptor expression on cell surface, cells were harvested by trypsinization at time points as specified in figure legends. The collected cells were incubated in 100 µl phycoerythrin (PE)-conjugated anti-DR4 or anti-DR5 antibodies (eBioscience) at RT for 30 min in dark. A PE-conjugated mouse IgG isotype control (eBioscience) was used as negative control. Fluorescence signals were then acquired on a FACSCalibur™ and analyzed as described above.

### Silencing gene expression with siRNAs and shRNA lentiviruses

SNU-216 was transiently transfected with the following siRNAs procured from Genolution Pharmaceuticals, Inc. (Seoul, Korea): DR5 siRNA (5′-AAGACCCTTGTGCTCGTTGTC-3′) and scramble siRNA as a control. Cells were transfected at 40 µM siRNAs using the Lipofectamine 2000^®^ transfection reagent (Life Technologies, Carlsbad, CA, USA) as instructed in the manufacturer's protocol. Cells were treated with TRAIL and/or bortezomib as specified in each experiment at 48 h after transfection. The pLKO.1 lentiviral vector with a scramble sequence, p21^cip1/waf1^ shRNA or DR5 shRNA (all from Sigma-Aldrich) were transfected into HEK293 cells by calcium phosphate precipitation. After 48h of transfection, viral supernatant was collected and filtered through 0.45 µm strain and stored at -80^°^C. SNU-216 was transduced with corresponding lentivirus in the presence of 8 µg/ml polybrene (Sigma-Aldrich). Infected cells were then selected by using medium containing 2.5 µg/ml of puromycin for 7 days before performing further experiments.

### Gene expression analysis

Gene expression data in median *z* scores from RNASeq V2 RSEM of 478 gastric cancer patients with clinical information was obtained from TCGA via cBioPortal for Cancer Genomics (http://www.cbioportal.org/, 12, 13). Kernel density plot, Shapiro-Wilk normality test, Wilcoxon rank sum test, Kruskal-Wallis rank sum test, Kaplan-Meyer survival analysis and log rank test were carried out with R statistical computing software (https://www.r-project.org/). Correlation coefficients among expression of TRAIL receptors and TRAIL were calculated by CORREL implemented in Microsoft Excel.

### Statistical analysis

All of the data are shown either as the mean 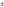 standard error of deviation (SE) or as the mean 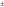 standard deviation (SD) from at least three independent experiments. Statistical comparison was performed with two-tailed Student's *t*-test or one-way ANOVA with *post hoc* Turkey's test. The *p* value smaller than 0.05 was considered statistically significant. The combination index (CI) was determined by using Compusyn software (http://www.combosyn.com/) in order to analyze the cooperation between TRAIL and bortezomib regarding to synergism, additivity, and antagonism 14, 15.

## Results

### Sensitivity of six gastric cancer cell lines to TRAIL

**Figure 1:**
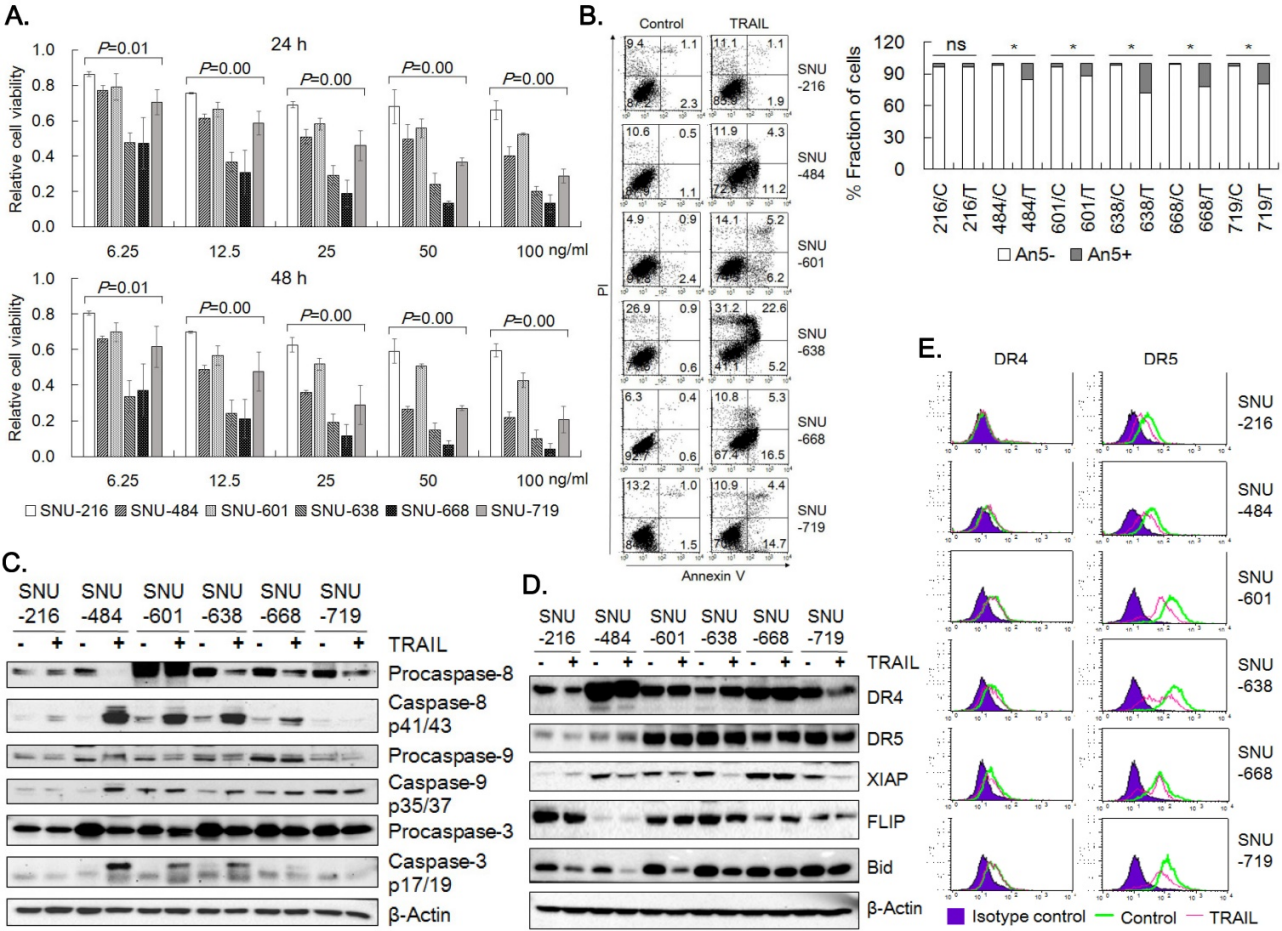
** TRAIL-induced apoptosis of six gastric cancer cell lines.** (A) TRAIL-induced reduction of cell viability of six gastric cancer cell lines. Cells were seeded in 96-well plates one day prior to TRAIL treatment at 0, 6.25, 12.5, 25, 50 and 100 ng/ml for 24 h and 48 h. Cell viability was assessed by MTT assay and relative cell viability was calculated against untreated control. Data shown is the mean ± standard error (SE) of three independent experiments. *P* values were calculated from one-way ANOVA. (B) Six gastric cancer cell lines were treated with TRAIL at 25 ng/ml for 24 h, and then TRAIL-induced apoptosis was determined by flow cytometric analysis of Annexin V/PI binding. Right panel shows fractions of Annexin V positive and negative cells, respectively. * for *P*<0.05 calculated by Student's *t*-test. (C) Activation of caspases-3, -8 and -9 was examined by detection of procaspases and active caspase fragments in western blot analysis with whole-cell lysate preparation from indicated cell lines treated with TRAIL at 25 ng/ml for 24 h. (D) Expression of death receptors and apoptosis modulators in untreated and treated cells with TRAIL (25 ng/ml) for 24 h were examined by western blot analysis*.* (E) Surface expression of DR4 and DR5 in untreated and treated cells with TRAIL (25 ng/ml) for 24 h were analyzed by flow cytometry. Results shown are representatives of three independent experiments (B, C, D and E).

Six gastric cancer cell lines were treated with increasing concentration of TRAIL and cell viability was measured by MTT assay at 24 h and 48 h (Fig. 1A). Sensitivity to TRAIL ranked in the order of SNU-668 and SNU-638 > SNU-719, SNU-484 and SNU-601 > SNU-216. Cell viability of the SNU cells except SNU-216 was decreased in a dose-dependent manner up to 100 ng/ml TRAIL, while the viability of SNU-216 was decreased up to 25 ng/ml TRAIL, but not significantly reduced further in 50~100 ng/ml TRAIL range. TRAIL-induced apoptosis of the gastric cancer cells was further validated by analyzing Annexin V binding with flow cytometry and caspase activation with western blotting. TRAIL treatment significantly increased Annexin V-positive cell fractions in the gastric cancer cells except SNU-216 (Fig 1B). The cleaved caspases-8, -9, and -3 were evidently detected in all cell lines, again except SNU-216 (Fig 1C). Obviously, TRAIL induced apoptotic cell death at varying extent in the six gastric cancer cells, and SNU-216 was found the most resistant among them.

In order to identify molecular determinants of the differential sensitivity, we analyzed the expression of molecules in the apoptotic signaling pathway of TRAIL by western blotting (Fig. 1D). DR4 expression was detected in all six cell lines, but lowest in SNU-216. DR5 expression was also evident, but the expression level was lower in SNU-484 and SNU-216. Basal XIAP level was lowest in SNU-216 and decreased upon TRAIL treatment in all cells, whereas FLIP expression was highest in SNU-216 and lowest in SNU-484. Bid expression varied among the cells, but decrease in full length Bid upon TRAIL treatment was observed in all six cells. The expression of DR4 and DR5 was evaluated in the six gastric cancer cells by flow cytometry (Fig. 1E). Flow cytometry verified comparable DR4 expression in all cells except SNU-216. DR5 expression was detected in all six cell lines by flow cytometry, but low in SNU-216 and SNU-484 as was shown in the western blotting (Fig. 1D). Reduction in surface DR5 expression upon TRAIL treatment was obvious in all six cells, while surface DR4 level noticeably decreased only in SNU-638 and SNU-668 upon TRAIL treatment. Collectively, expression levels of DR4, DR5 and FLIP might be associated with observed high resistance of SNU-216 against TRAIL.

### Gene expression analysis of DR4 and DR5 in gastric cancer tissue

**Figure 2:**
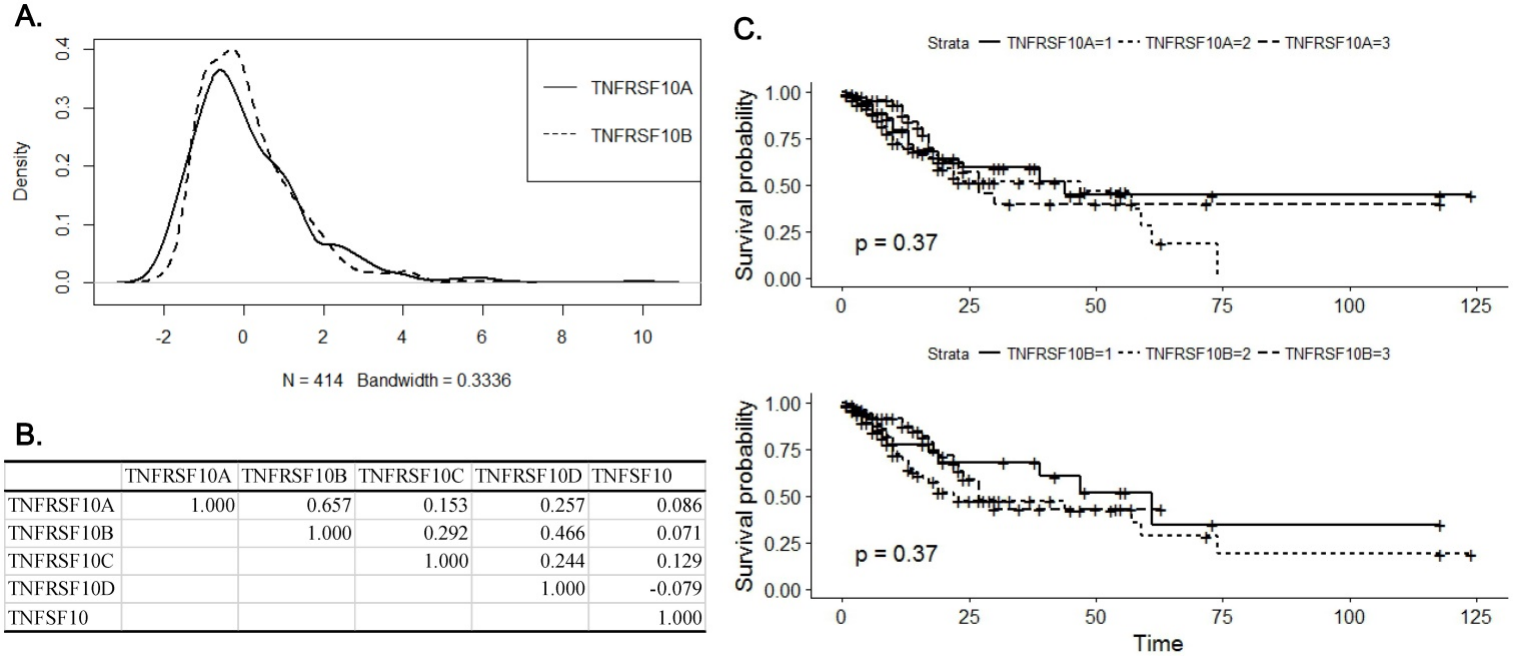
** Analysis of DR4 and DR5 expression in gastric cancer tissue with RNASeq results in TCGA database.** (A) A Kernel density plot represents distribution of median *z*-score of DR4 (TNFRSF10A) and DR5 (TNFRSF10B) expression in gastric cancer tissue. (B) A table of correlation coefficients among DRs, DcRs (TNFRSF10C and TNFRSF10D) and TRAIL (TNFSF10). (C) Kaplan-Meier plots for overall survival according to expression of DR4 (upper panel) and DR5 (lower panel). Solid lines represent for expression lower than the first quartile, dotted lines for expression between the first and third quartile and dashed lines for expression higher than the third quartile, respectively.

Resistance to TRAIL-induced apoptosis of gastric cancer cells appeared to be associated with the expression of apoptotic TRAIL receptors. Therefore, we analyzed the expression of DR4 and DR5 in gastric cancer tissue using RNASeq results in TCGA database. Median *z*-values were distributed between -2.19~9.90 for DR4 and -2.07~6.57 for DR5, respectively (Fig. 2A). Expression of DR4 correlated with that of DR5 with correlation coefficient of 0.657, which was highest among all 18,447 genes included in the analysis (Fig. 2B). Correlation coefficients of DR4 or DR5 expression with TRAIL and TRAIL decoy receptors were less than 0.5. DR4 expression was significantly higher in early stage tumor with low infiltration and no nodal involvement (p=0.014, p=0.015 and p=0.018 for T stage, N stage and TNM stage, respectively, Table 1). In contrast, expression of DR5 was marginally associated with N stage and grade (p=0.064 and p=0.065, respectively) and was significantly higher in intestinal type than diffused type (p=0.018). However, survival analysis with Kaplan-Meier estimation showed that either DR4 or DR5 expression did not significantly affect overall survival (p=0.37 for DR4 and p=0.37 for DR5, respectively (Fig. 2C). Expression of DR4 and DR5, especially DR4, appears to be decreased with progression of gastric cancer at early stage, but not with development of metastatic capacity, which might explain the observed irrelevance of patient survival with DR4 and DR5 expression.

### Combined treatment of TRAIL with bortezomib

**Figure 3:**
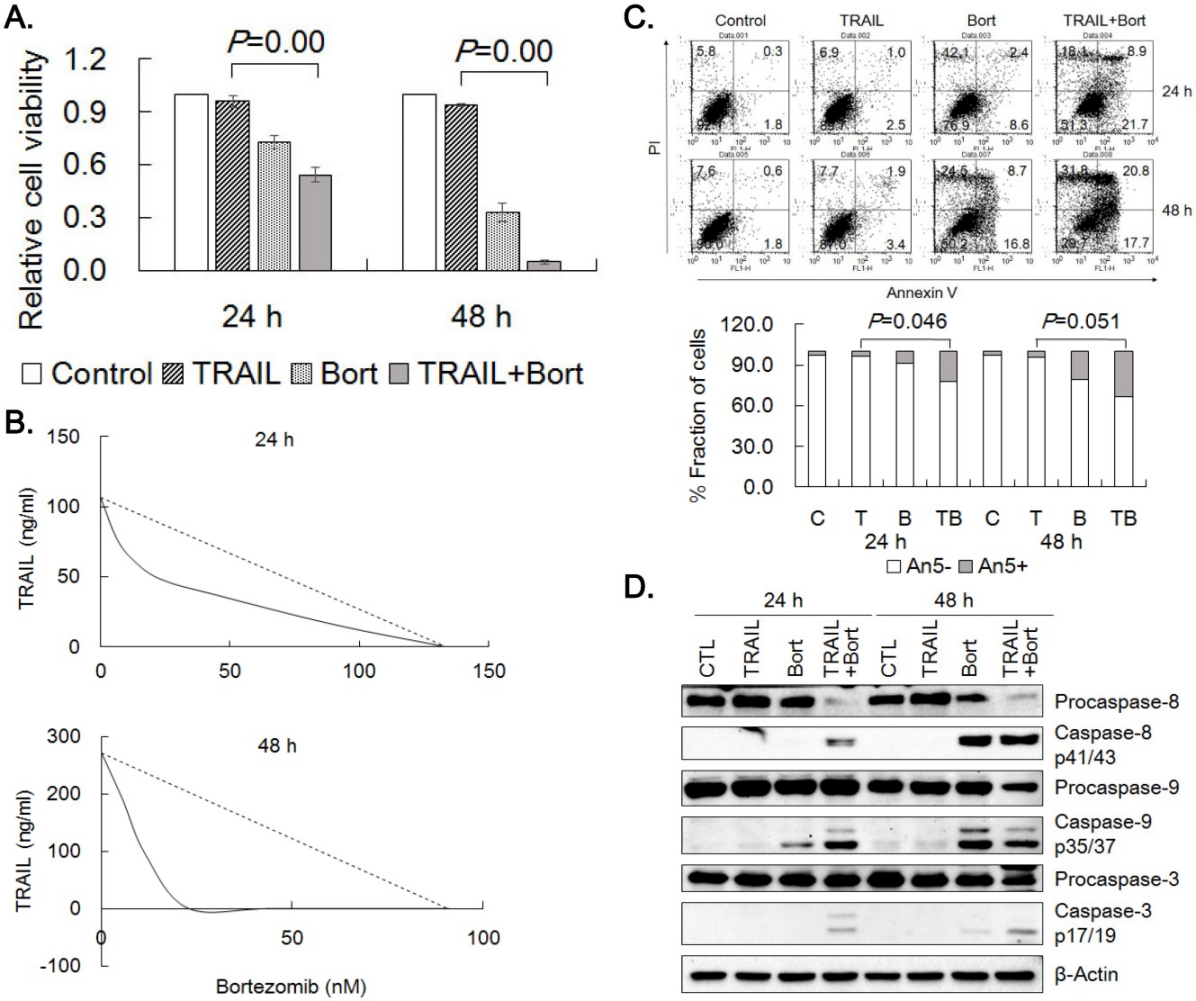
** Synergistic effects of TRAIL and bortezomib in SNU-216.** (A) SNU-216 was treated with TRAIL (12.5 ng/ml) in combination with bortezomib (22.75 nM) for 24 h and 48 h. Cell viability was analyzed by MTT assay and relative cell viability was calculated against untreated control. Results are presented as the mean ± SE of three independent experiments. *P* values were calculated from one-way ANOVA. (B) The cells were co-treated with TRAIL (0.0, 12.5, 25.0 and 50 ng/ml) and bortezomib (0.0, 5.7, 11.4, 22.8, 45.5 and 91.0 nM) in a range of concentration for 24 h and 48 h. Cell viability was measured by MTT assay and synergism of drug combinations was analyzed with isobologram. (C) Apoptotic cells were determined by flow cytometry of Annexin V/PI staining. Lower panel shows fractions of Annexin V positive and negative cells, respectively.* P* values were calculated from one-way ANOVA. (D) Western blot was used to detect the activation of caspases-3,-8 and -9. The data shown are representatives of triple experiments (C and D).

In order to potentiate the efficacy of TRAIL, we treated the gastric cancer cells with 20 different known and putative cancer therapeutics plus TRAIL at single concentration combinations and evaluated their ability to enhance TRAIL cytotoxicity. Four reagents including proteasome inhibitors (bortezomib and MG132) and anthracyclines (doxorubicin and daunorubicin) were found to potentiate cytotoxicity of TRAIL more than expected by simple multiplication of individual drug effect. Since bortezomib enhanced TRAIL cytotoxicity, the effect of bortezomib on TRAIL was further characterized in TRAIL-resistant SNU-216 cells. Viable cell amount was significantly decreased when combined treatment of TRAIL and bortezomib was compared with that of TRAIL or bortezomib alone (Fig. 3A). IC_50_-equivalent amount of TRAIL at various TRAIL/bortezomib concentration combinations in SNU-216 were located well below the line of additivity in isobolograms, indicating strong synergistic interaction at all combinations (Fig. 3B). TRAIL-induced apoptosis of SNU-216 was further analyzed by Annexin V binding after 24 h and 48 h treatment of TRAIL alone or in combination with bortezomib. In accordance with the MTT results, combined treatment of TRAIL and bortezomib significantly increased Annexin V positive fractions over TRAIL treatment alone (Fig. 3C). In addition, TRAIL/bortezomib enhanced the activation of caspases-8, -9 and -3 (Fig. 3D). Collectively, these results clearly demonstrated that bortezomib could synergize TRAIL-mediated apoptosis of the gastric cancer cells.

### Bortezomib upregulates p21^cip1/waf1^

**Figure 4:**
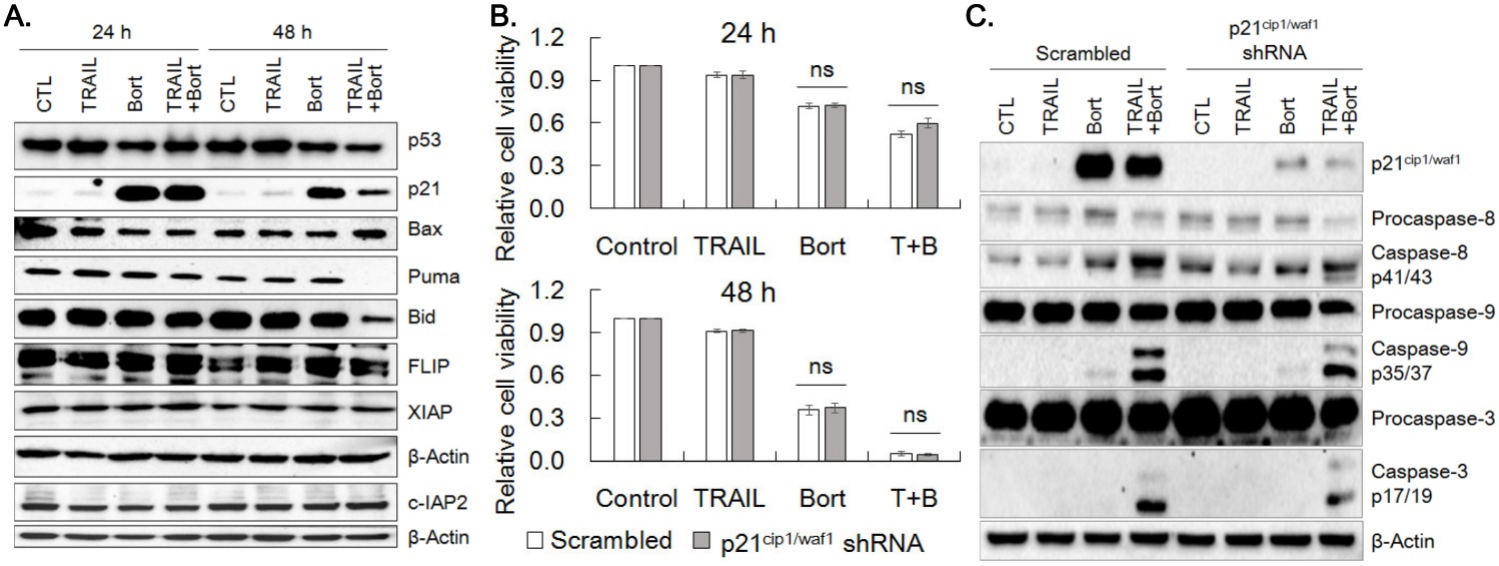
** Bortezomib upregulates p21^cip1/waf1^.** (A) SNU-216 cells were treated with TRAIL and/or bortezomib for 24 h and 48 h. The expression of indicated proteins including p21^cip1/waf1^ were visualized by western blot analysis. (B) SNU-216 cells were transduced with scramble or p21^cip1/waf1^ shRNA expressing lentiviruses, then were selected by puromycin (2.5 µg/ml) for one week. The p21^cip1/waf1^ knockdown cells were treated with TRAIL in the presence or absence of bortezomib for 24 h and 48 h. Cell viability of SNU-216 after 24 h and 48 h treatment was measured by MTT assay and relative cell viability was calculated against untreated control. MTT results shown are the means ± SE of three independent experiments. 'ns' for *P*>0.05 in Student's *t*-test. (C) Expression of p21^cip1/waf1^ and activation of caspases in the p21^cip1/waf1^ knockdown cells were visualized by western blot analysis. All cells (A~C) were treated with TRAIL at 12.5 ng/ml and/or bortezomib (22.75 nM) for indicated time period. The images shown are representatives of triple experiments (A and C).

To understand the mechanism underlying the synergistic effect of bortezomib on TRAIL, we examined the expression of proteins modulating cell proliferation and apoptosis. p21^cip1/waf1^ expression was markedly increased by 24 h and 48 h treatment of bortezomib and bortezomib plus TRAIL, whereas expression of p53, Bid and Puma was decreased by bortezomib plus TRAIL treatment at 48 h noticeably (Fig. 4A, Supplemental Fig. 1). However, bortezomib did not significantly alter protein levels of Bax, FLIP, cIAP2 and XIAP. To determine whether bortezomib-enhanced TRAIL sensitivity was ascribed to increased expression of p21^cip1/waf1^, expression of p21^cip1/waf1^ was knocked down with p21^cip1/waf1^ shRNA and susceptibility of cells to the combined treatment was examined. Cell viability of the p21^cip1/waf1^-knockdown cells were insignificantly increased at 24 h and 48 h after treatment of bortezomib alone or TRAIL/bortezomib, compared to that of scramble shRNA expressing cells (Fig. 4B). However, activation of caspases-3, -8, and -9 upon combined treatment of TRAIL and bortezomib was mitigated in the p21^cip1/waf1^-knockdown cells than in scramble shRNA expressing ones (Fig. 4C), which appears to reflect marginal increase in viability of p21^cip1/waf1^-knockdown cells treated with TRAIL plus bortezomib for 24 h (Fig. 4B). These results suggested that accumulation of p21^cip1/waf1^ by bortezomib contributes to the enhanced TRAIL-induced apoptosis of SNU-216 cells insignificantly.

### Overexpression of DR5 by bortezomib

**Figure 5:**
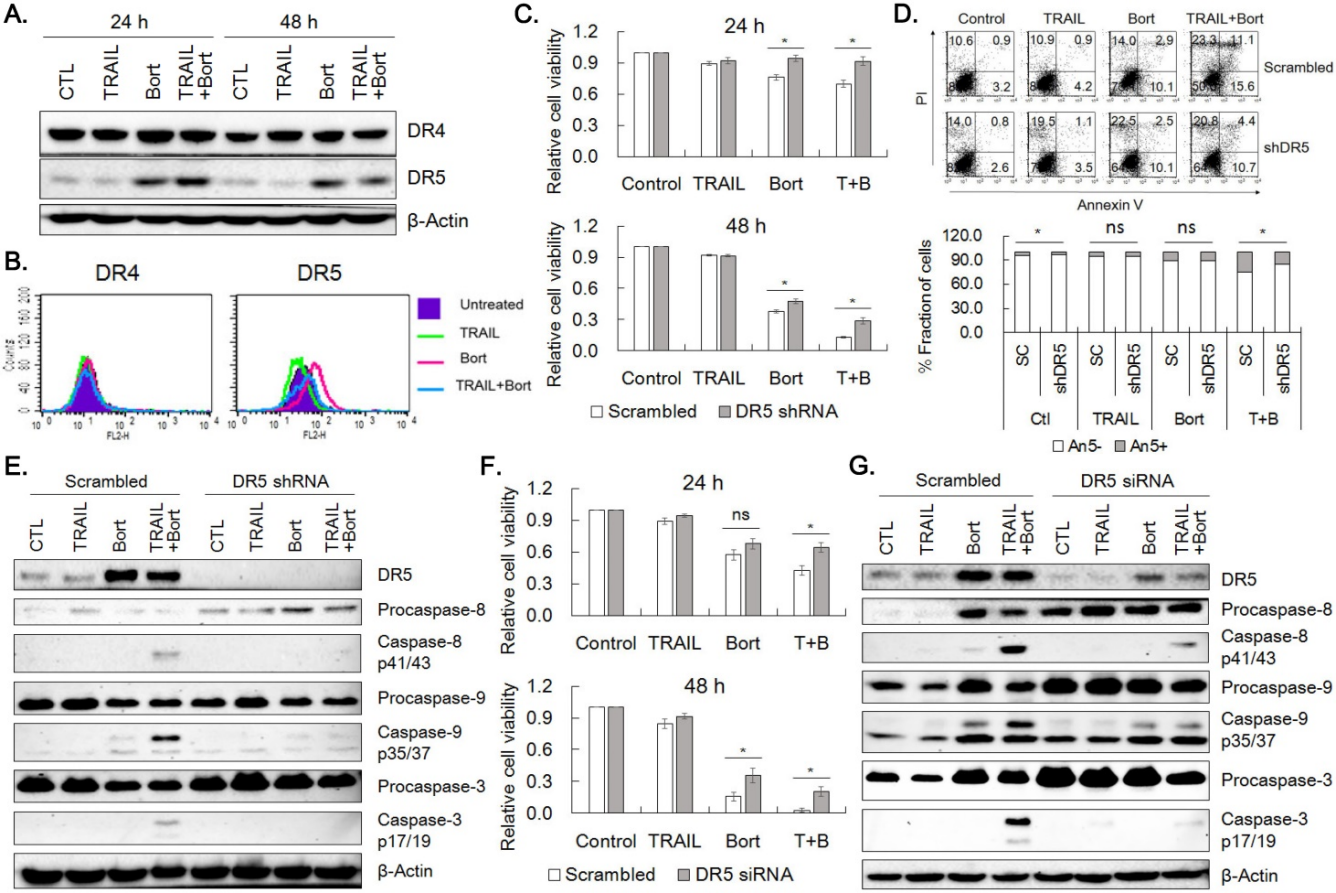
** DR5 contributes to bortezomib-induced TRAIL sensitization.** (A) Effects of bortezomib on the expression of DR4 and DR5 in SNU-216. Cells were treated with TRAIL and/or bortezomib for 24 h and 48 h, and expression of DR4 and DR5 was examined by western blot analysis. (B) Surface expression of DR4 and DR5 in SNU-216 upon 24 h expose to TRAIL in the presence or absence of bortezomib, was analyzed by flow cytometry. (C) Lentivirus of scramble control or DR5 shRNA was transduced into SNU-216, and then selected with puromycin (2.5 µg/ml) for a week. Cell viability of scramble and DR5-silenced cells upon TRAIL and/or bortezomib treatment for 24 h and 48 h was measured by MTT assay and relative cell viability was calculated against untreated control. Results shown are means ± SE of three independent experiments. (D) Reduced Annexin V binding in the DR5-silenced cells treated with TRAIL and/or bortezomib was analyzed by flow cytometry. Lower panel shows fractions of Annexin V positive and negative cells, respectively. (E) Expression of DR5 and activation of caspases in the DR5-silenced cells treated with TRAIL and/or bortezomib were analyzed by western blot. (F) Expression of DR5 in SNU-216 was knocked down by transfection of DR5 siRNA. DR5-silenced cells by siRNA transfection were treated with TRAIL and/or bortezomib for 24 h and 48 h and cell viability was measured by MTT assay. Relative cell viability was calculated against untreated control and shown are the means ± SE of three independent experiments. (G) Expression of DR5 and activation of caspases in the DR5-silenced cells were examined by western blot analysis. The siRNA transfected cells were treated with TRAIL and/or bortezomib for 24 h. All cells (A~G) were treated with TRAIL at 12.5 ng/ml and/or bortezomib (22.75 nM) for indicated time period. * for *P*< 0.05 and 'ns' for *P*>0.05 calculated from Student's *t*-test (C, D and F). Results shown are representatives of triple experiments (A, B D, E and G).

Since accumulation of p21^cip1/waf1^ was not enough to explain viability reduction observed in the combined treatment, the effect of combined treatment of TRAIL and bortezomib on the expression of DR4 and DR5 was examined in SNU-216 cells. Bortezomib increased expression of DR5 obviously and DR4 to a lesser extent at 24 h and 48 h of the drug treatment (Fig. 5A). Consistently, flow cytometric analysis for the receptors confirmed that the expression of DR5 was increased in SNU-216 treated with bortezomib alone or in combination with TRAIL (Fig. 5B). These observations implied that upregulation of DR5 might play a critical role in sensitization of TRAIL-induced apoptosis of SNU-216 by bortezomib. In order to assess the involvement of DR5 in sensitization of TRAIL-induced apoptosis by bortezomib, we knocked down DR5 expression by shRNA. Knockdown of DR5 significantly increased cell viability when compared with scramble control in both 24 h and 48 h of TRAIL and bortezomib treatment (Fig. 5C). Of notice, cell viability of DR5-silenced cells treated with TRAIL plus bortezomib was comparable with that of scramble control treated with bortezomib alone. In addition, Annexin V-positive fraction of the DR5-silenced cells was reduced by 38% in average when compared with that of scramble control upon combined treatment of TRAIL and bortezomib for 24 h (*p*=0.03, Fig. 5D). The activation of caspases-8, -9 and -3 was also reduced in DR5-silenced cells when compared with scramble control (Fig. 5E). Furthermore, silencing DR5 expression by siRNA transfection also resulted in comparable viability increase shown in DR5 shRNA expressing cells and decreased activation of caspases -8, -9 and -3 (Fig. 5F and 5G, respectively). Taken together, upregulation of DR5 by bortezomib was supposed to be a critical factor for the TRAIL-bortezomib synergy.

### ERK1/2 activity is required in the upregulation of DR5 by bortezomib

**Figure 6:**
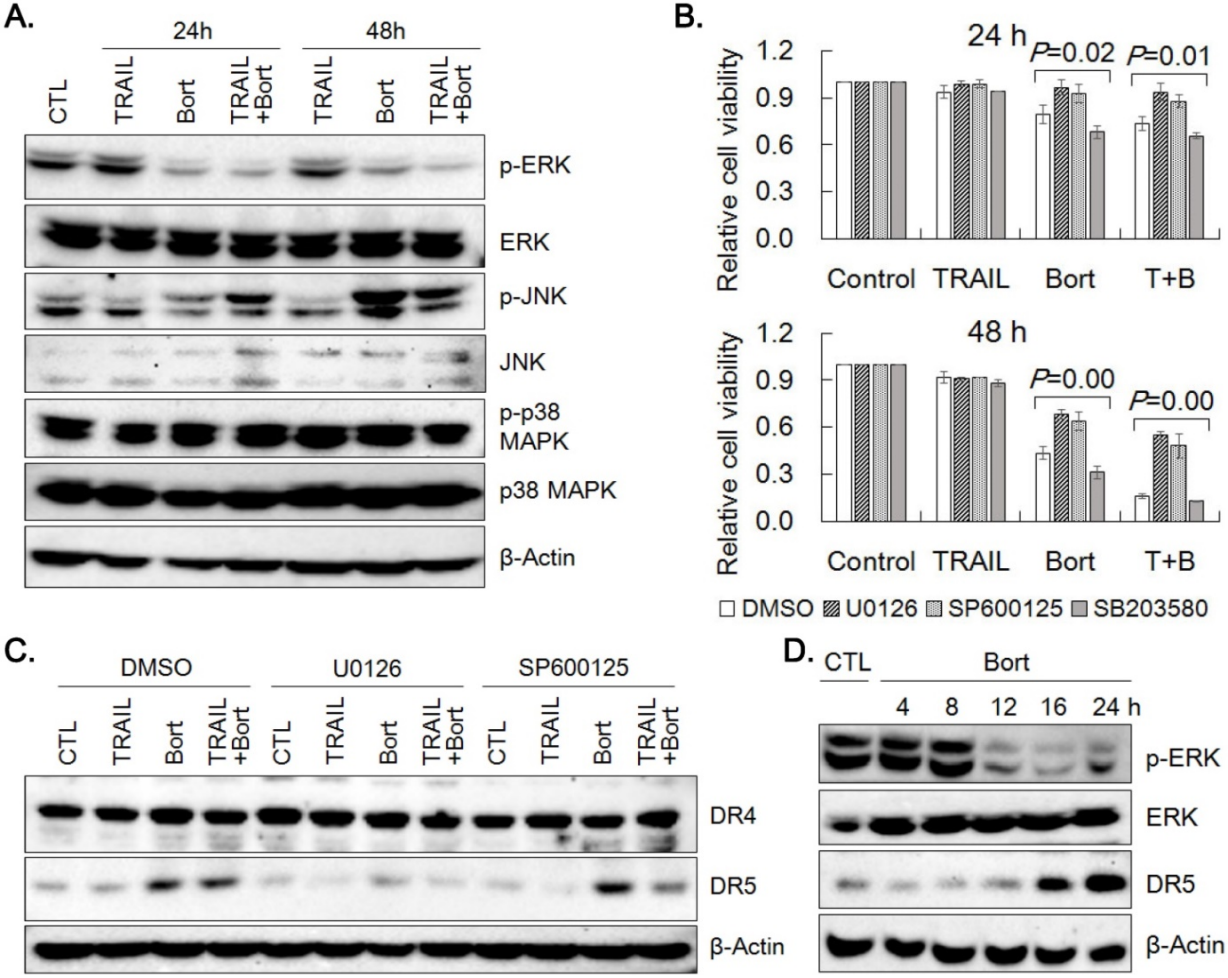
** Activation of MAPK signaling pathway by bortezomib.** (A) Activation of ERK1/2, JNK and p38 MAPK in SNU-216 treated with TRAIL and/or bortezomib for 24 h and 48 h was analyzed by western blotting with antibodies against activation-associated phosphorylation of the kinases. (B) Cells were pretreated with U0126 (20 µM), SP600125 (20 µM) or SB203580 (20 µM) for 1 h followed by TRAIL and/or bortezomib treatment for 24 h and 48 h. Cell viability was measured by MTT assay and relative cell viability was calculated against untreated control. Results shown are the means ± SE of three independent experiments. *P* values were calculated from one-way ANOVA. (C) Expression of DR4 and DR5 in SNU-216 pretreated with U0126 (20 µM) or SP600125 (20 µM) for 1 h followed by TRAIL and/or bortezomib treatment for 24 h was visualized by western blot analysis. (D) Time-dependent expression of DR5, phospho-ERKs and ERKs in cells treated with bortezomib was examined by western blot analysis. All cells (A~D) were treated with TRAIL at 12.5 ng/ml and/or bortezomib (22.75 nM) for indicated time period. The data shown are representatives of triple experiments (A, C and D).

Since the overexpression of DR5 was found critical in TRAIL and bortezomib synergism, we attempted to explore mechanism by which bortezomib could upregulate DR5 expression. DR5 expression is known to be modulated by ERK, JNK and p38 MAPK 16, 17. Hence, the effect of TRAIL and bortezomib on expression and phosphorylation of the MAPKs was examined by western blotting (Fig. 6A). Bortezomib reduced phosphorylation of ERK1/2, but increased that of JNK, and did not alter that of p38 MAPK without significant changes in the expression level of the kinases. Reduction of cell viability caused by treatment of bortezomib and bortezomib plus TRAIL was significantly recovered by pretreatment of SNU-216 with U0126 (an inhibitor of ERK1/2 activation) and SP600125 (a JNK inhibitor), but not with SB203580 (a p38MAPK inhibitor) (Fig. 6B). The effects of U0126 and SP600125 on the expression of DR4 and DR5 was determined by western blotting. Whereas DR4 expression remained unchanged upon pretreatment of U0126 and SP600125, upregulation of DR5 level by bortezomib was dampened significantly only by U0126 pretreatment, but not by SP600125 (Fig. 6C, Supplemental Fig. 2). Since DR5 upregulation by bortezomib appeared to be associated with ERK activation, time-dependent expression of phospho-ERKs and DR5 was examined by western blotting. The level of phospho-ERKs was maintained until 8 h, but dropped in 12 h, whilst DR5 level was increased after 16 h of bortezomib treatment (Fig. 6D). These results suggest that upregulation of DR5 expression by bortezomib might be dependent on ERK1/2 activity at early time point.

## Discussion

The correlation of TRAIL sensitivity with the expression of TRAIL receptors and their immediate signal modulators was examined in six human gastric cancer cell lines. SNU-216 in which level of DR4 and DR5 was lowest, but that of FLIP_L_ was highest among them, was found the most resistant to TRAIL. Correlation of death receptor expression with TRAIL sensitivity was also found in SNU-1 gastric cancer cells that showed negligible DR4 expression and manifested strong resistance against TRAIL 11. Although a critical role of various other modulators in TRAIL-induced apoptosis cannot be ruled out, therefore, surface expression level of DRs should be considered as a critical factor in TRAIL response of the gastric cancer cells. TRAIL resistance of SNU-216 might also be ascribed to high expression level of FLIP through constitutive activation of PI3K/AKT pathway 18, which suggests considerable significance of other apoptosis regulators in modulation of TRAIL response of the gastric cancer cells.

Association of death receptor expression with pathology of gastric cancer tissue at mRNA level revealed that DR4 expression was significantly higher in early stage tumor without distant metastasis. DR5 expression was also associated with nodal status with marginal significance. Although clinical association of DR expression at mRNA level in gastric cancer has not been established yet, negative DR4 protein expression was found to correlate with lower nodal status with marginal significance in gastric cancer 19. Aside from gastric cancer, DR4 expression correlated with more differentiated tumors and negative nodal status in an immunohistochemical study of breast cancer, while DR5 expression correlated with higher tumor grade, proliferative index, positive nodal status and reduced overall survival rate 20. On the contrary, DR5 expression was reported to be reduced in higher grade prostate cancer 21. Therefore, DR4 expression appeared to be negatively associated with phenotypes of progressed tumor, albeit correlation of DR5 expression with tumor grade and survival is controversial, yet.

In order to overcome TRAIL resistance observed in the gastric cancer cells, TRAIL cytotoxicity was examined in combination with various chemotherapeutics. Strong synergy in induction of apoptosis of the gastric cancer cells was observed by combined treatment of TRAIL with proteasome inhibitors (bortezomib and MG132) and anthracyclines (doxorubicin and daunorubicin). Bortezomib (VELCADE^®^) that was approved by the Food and Drug Administration (FDA) for the treatment of multiple myeloma is a selective 26S proteasome inhibitor 22, 23. Bortezomib elicits G_2_/M arrest and induces apoptosis of diverse cancer cells by itself and in combined treatment with various known and potential cancer therapeutics. Bortezomib-induced growth arrest and apoptosis are mediated by inhibition of NF-κB activation, increased expression of growth arresting and apoptotic proteins as well as induction of ER stress 24-26. Bortezomib also enhances the efficacy of TRAIL in several cancer cells including gastric cancer cells 27. Depending on the cellular context, bortezomib modulates the expression of TRAIL receptors, c-FLIP, Bik, Bim, IAPs, p21^cip1/waf1^ and p27^kip^, and activation of NF-κB, Akt and MAPKs, which has been suggested for potential mechanisms behind the synergy 22.

Treatment of bortezomib on TRAIL-resistant SNU-216 cells elicited G_2_/M arrest and apoptosis of the cancer cells. Bortezomib treatment significantly increased expression of p21^cip1/waf1^, DR5 and DR4 which could directly modulate cell cycle progression and apoptosis. Bortezomib is a proteasome inhibitor that can stabilize and increase p53 level 28. However, bortezomib by itself did not increase level of p53 and its targets including Bax and Puma in SNU-216 cells. Instead, expression of p53 and Puma was significantly decreased by combined treatment of TRAIL plus bortezomib for 48 h, suggesting that downregulation of p53 and Puma might result from massive cell death at late time point. On the other hand, expression of p21^cip1/waf1^ and DR5 was significantly increased in bortezomib-treated cells even without noticeable change in p53 at early time point. Oridonin, a herbal diterpenoid, increased expression of p53 and Bax in SNU-216 cells, demonstrating its inducibility and functionality in the cells 29. Therefore, it is conceivable that bortezomib might activate an alternative signaling pathway that obviates p53 induction to upregulate DR5 and p21^cip1/waf1^ expression. Activation of ATF4-ATF3/CHOP axis via PKCdelta 30 or change in ERK1/2 activity profile (Fig. 6) by bortezomib could be an alternative signaling pathway for induction of DR5. Indeed, bortezomib-induced DR5 expression is regulated by CHOP, an ER-stress mediator in several cells including human non-small cell lung cancer cells 31. Insignificant change in Bax and decrease in p53 and Puma in conjuction with seemingly p53-independent induction of DR5 and p21^cip1/waf1^ cast doubt on the role of p53 in the synergistic death of SNU-216 cells by combined treatment of bortezomib and TRAIL.

Upregulation of p21^cip1/waf1^ by bortezomib has been reported in many cancer cells, which might be associated with cell cycle arrest upon bortezomib treatment 32. Increase in p21^cip1/waf1^ and concomitant decrease in CDK activity by bortezomib are found responsible for sensitization of bladder and prostate cancer cells to TRAIL-induced apoptosis 33. However, silencing p21^cip1/waf1^ in SNU-216 cells marginally increased cell viability and decreased level of cleaved caspases upon treatment of bortezomib alone or TRAIL/bortezomib for 24 h. The rescuing effect of p21^cip1/waf1^ knockdown was too modest to support a determinative role of p21^cip1/waf1^ in the TRAIL/bortezomib synergy. Hence, although p21^cip1/waf1^ level is strongly increased by bortezomib treatment, the role of p21^cip1/waf1^ in the TRAIL/bortezomib synergy remains to be doubtful.

Liu *et al.* reported synergistic apoptosis of different gastric cancer cells by co-treatment of bortezomib and TRAIL 27. Increase in both DR4 and DR5 and decrease in c-IAP1 were observed in the SGC7901 gastric cancer cells. Increase in DR5 only or in both DR4 and DR5 was also reported in other cancer cells, which was considered as a critical component in TRAIL/bortezomib synergy 27, 31. In accord, silencing DR5 expression in SNU-216 by shRNA expression or siRNA transfection increased cell viability of combined treatment of TRAIL and bortezomib up to ~80% of bortezomib single treatment (Fig. 5). Thus, upregulation of DR5 by bortezomib was mainly responsible for bortezomib synergy in TRAIL-induced apoptosis, as was reported in many other cancer cells 31, 34.

How bortezomib modulates the expression of DR5 has not been fully understood. Activation of the MAPKs including ERKs, JNK and p38 MAPK was known to stimulate DR5 expression in many cancer cells 17, 35, 36. In SNU-216, phospho-ERK1/2 was decreased, but phospho-JNK was increased by 24 h treatment of bortezomib. Both ERK and JNK inhibitors were able to counteract the cytotoxicity of bortezomib only and TRAIL/bortezomib treatment. However, inhibition of ERK1/2 activation, not JNK inhibition attenuated bortezomib-induced DR5 upregulation. Collectively, although both ERK and JNK pathway in parallel contribute to synergistic apoptosis by TRAIL and bortezomib, DR5 expression was regulated in ERK1/2-dependent manner.

Upregulation of DR5 was associated with increased phosphorylation of ERK1/2 in bortezomib-treated lung cancer cells 31. However, bortezomib was also known to decrease phosphorylation of ERK1/2 via a MAPK phosphatase-3-dependent pathway in transformed endothelial cells 37. In SNU-216, phospho-ERK level was significantly reduced after 8 h treatment of bortezomib, whereas increased DR5 expression was detected after 16 h of treatment (Fig. 6D). Apparently, these results suggest that upregulation of DR5 could result from reduction of phospho-ERKs. Surprisingly, however, inhibition of ERK1/2 by U0126 prevented either basal or induced level of DR5 expression from increasing and partially reverted cell viability reduction upon bortezomib treatment, which argues for the role of ERKs in the process. In reconciliation, presence of active ERKs at the early stage of bortezomib treatment appears to be required for bortezomib-induced cytotoxicity and DR5 upregulation. However, mode and mechanism of action of ERKs in the bortezomib-induced changes remains to be elucidated in detail.

## Conclusion

Gastric cancer cells varied in response to TRAIL, which might be explained by differential expression of DR4 and FLIP. Although expression of DR4 and DR5 did not influence overall survival rate of gastric cancer patients, higher DR4 expression was found to correlate with lower T, N and TNM stages. TRAIL resistance of SNU-216 could be efficiently overcome by combined treatment of TRAIL and bortezomib. Upregulation of DR5 by bortezomib was found to contribute significantly to the TRAIL/bortezomib synergy. Both JNK and ERK1/2 were involved in cytotoxic effect of bortezomib and TRAIL/bortezomib treatments. However, only ERK1/2 activity at early time point of bortezomib treatment was required for the upregulation of DR5. Bortezomib also enhances the efficacy of TRAIL against tumor xenografts and endogenous cancers in animal studies 38. In addition, TRAIL is implicated in antitumor effect of NK cells which can be potentiated by bortezomib treatment 39. Thus, these results would provide important information on the utility of TRAIL as a therapeutic measure of gastric cancer with synergistic agents including bortezomib.

## Supplementary Material

Supplementary figures.

## Figures and Tables

**Table 1:** Association of clinicopathological parameters and death receptor expression in gastric cancer.

			TNFRSF10A				TNFRSF10B			
		No	1st Q	Median	3rd Q	*p value*	1st Q	Median	3rd Q	*p value*
Sex	Female	147	-0.713	-0.064	1.074	**0.047**	-0.748	0.002	0.815	0.096
	Male	267	-0.892	-0.325	0.655		-0.803	-0.261	0.527	
Age	<65	171	-0.801	-0.163	0.871	0.466	-0.807	-0.155	0.643	0.685
	>=65	234	-0.853	-0.211	0.805		-0.796	-0.115	0.688	
Histology	Intestinal	176	-0.849	-0.205	0.727	0.581	-0.613	-0.089	0.671	**0.018**
	Diffused	69	-0.851	-0.209	0.532		-0.932	-0.311	0.301	
Grade	G1/G2	159	-0.756	-0.097	0.763	0.358	-0.552	-0.119	0.859	0.065
	G3	246	-0.873	-0.321	0.822		-0.863	-0.158	0.568	
T status	T1/T2	109	-0.678	0.152	1.115	**0.014**	-0.803	-0.033	1.232	0.237
	T3/T4	296	-0.892	-0.291	0.665		-0.801	-0.158	0.534	
N status	N0	122	-0.693	0.068	1.115	**0.015**	-0.772	-0.011	0.998	0.064
	N1/N2/N3	273	-0.921	-0.341	0.668		-0.836	-0.195	0.534	
M status	M0	367	-0.871	-0.221	0.846	0.827	-0.801	-0.154	0.671	0.834
	M1	27	-0.660	-0.176	0.259		-0.686	0.010	0.615	
Tumor stage	Stage1/2	179	-0.790	0.096	1.047	**0.018**	-0.769	-0.061	0.882	0.262
	Stage3/4	210	-0.921	-0.333	0.572		-0.844	-0.156	0.477	

## Abbreviations

TRAIL: tumor necrosis factor-related apoptosis-inducing ligand
DR: death receptor
FLIP: FADD-like IL-1β-converting enzyme(FLICE)-inhibitory protein
IAP: inhibitor of apoptosis
XIAP: X-linked inhibitor of apoptosis
MAPK: mitogen-activated protein kinase
ERK: extracellular signal-regulated kinase
JNK: Jun N-terminal kinase
CDK: cyclin-dependent kinase
NK: natural killer
NF-kB: nuclear factor kappa-light-chain-enhancer of activated B cells
PI-3K: phosphoinositide 3-kinase
TCGA: The Cancer Genome Atlas
MTT: 3-(4,5-dimethylthiazol-2-yl)-2,5-diphenyltetrazolium bromide (MTT)
PI: propidium iodide
