# Supplementary material for: Synergistic apoptosis of human gastric cancer cells by bortezomib and TRAIL
Source: Int J Med Sci. 2019 Sep 20;16(11):1412–23. doi: 10.7150/ijms.34398 (PMC6818207; doi:10.7150/ijms.34398)
Supplement: Supplementary file 1 — Supplementary figures. [file ijmsv16p1412s1.pdf]

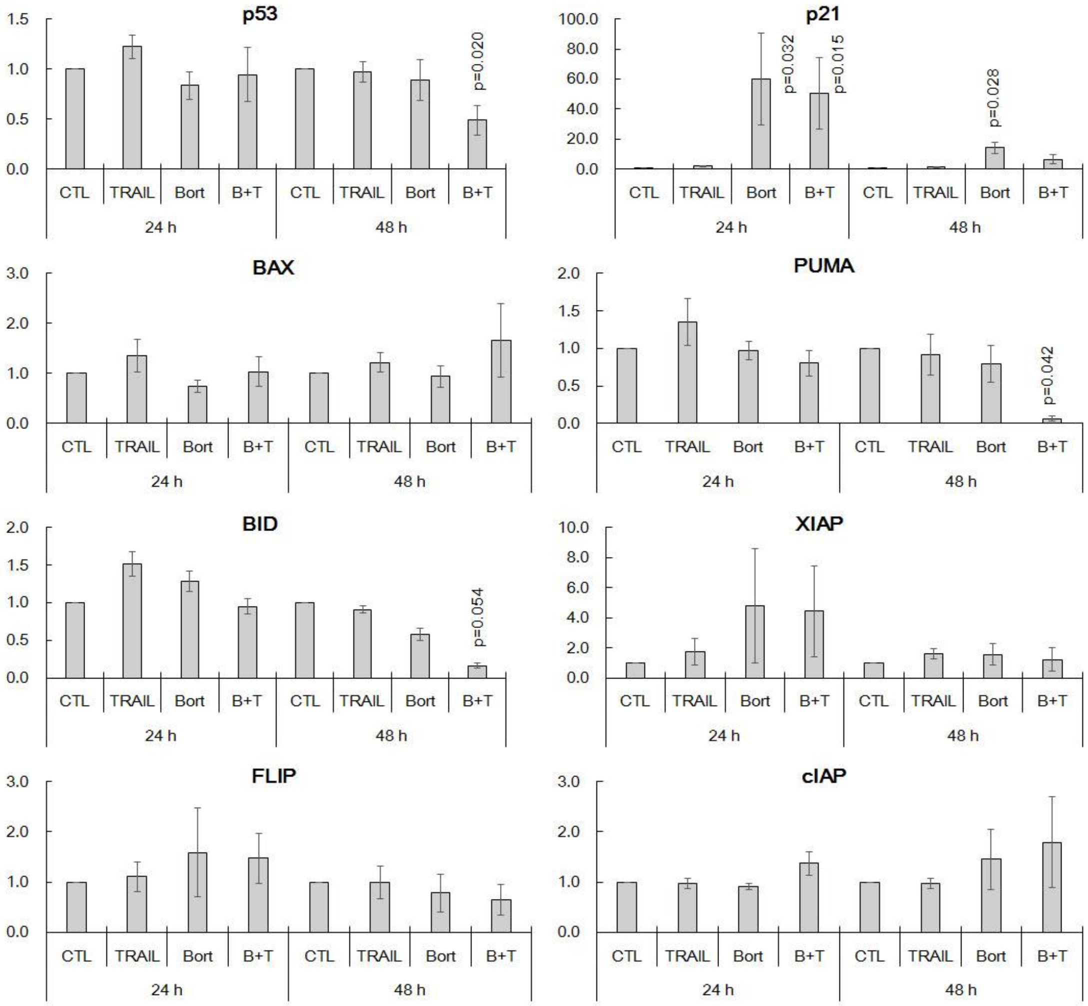

Supplemental Figure 1. Quantification of western blot results of Fig. 4A. Specific protein bands detected by western blots were quantified with Image J software and normalized against β-Actin. Data shown are means ± SE of more than triple experiments except duplicated Puma. Vertical axis represents fold changes of the normalized intensity against 24 h and 48 h controls, respectively. Statistical analysis was performed with Student's t-test between untreated control vs. each treat group. Bort for bortezomib; B + T for bortezomib plus TRAIL.

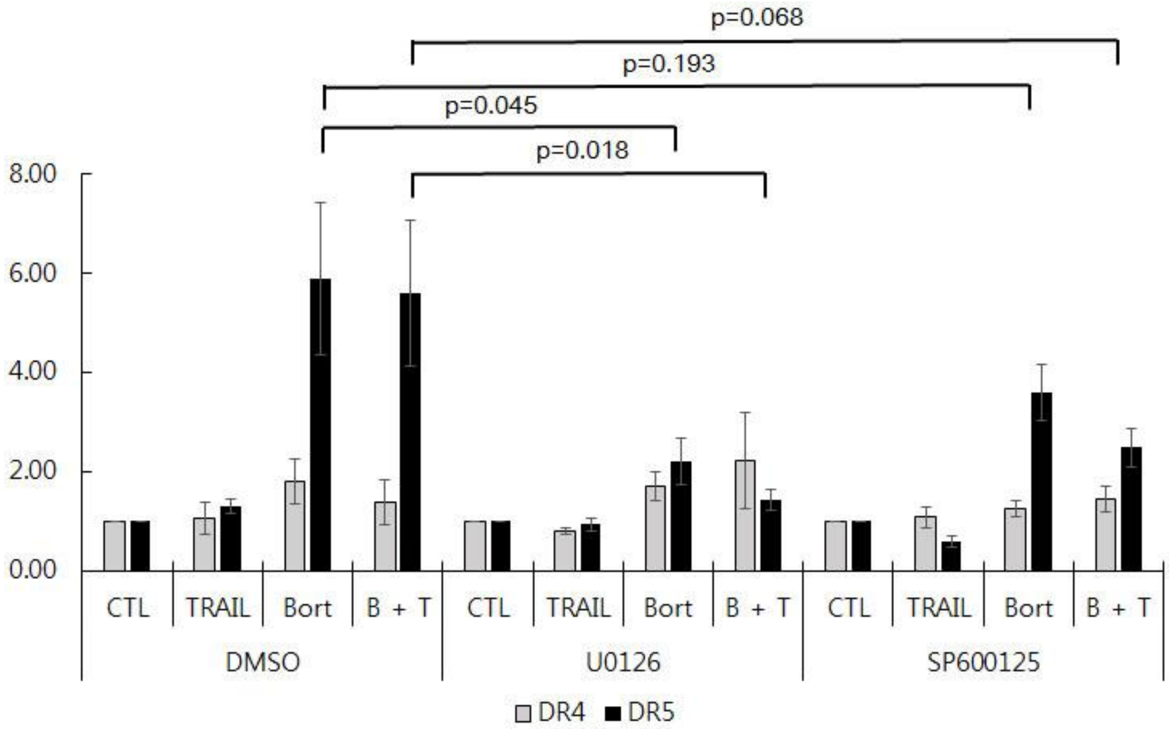

Supplemental Figure 2. Quantification of western blot results of Fig. 6C. Specific protein bands detected by western blots were quantified with Image J software and normalized against β-Actin. Data shown are means ± SE of more than quadruple experiments. Vertical axis represents fold changes of the normalized intensity against drug controls, respectively. Statistical analysis was performed with Student's t-test between untreated control vs. U0126 or SP600125-treated group independently. Bort for bortezomib; B + T for bortezomib plus TRAIL.
